# Expression of P-gp in acute myeloid leukemia and the reversal function of As_2_O_3_ on drug resistance

**DOI:** 10.3892/ol.2014.2692

**Published:** 2014-11-10

**Authors:** FENG GAO, WANWEI DONG, WEI YANG, JIA LIU, ZHIHONG ZHENG, KAILAI SUN

**Affiliations:** 1 Department of Genetics, China Medical University, Shenyang, Liaoning 110001, P.R. China; 2 The First Hospital of China Medical University, Shenyang, Liaoning 110001, P.R. China; 3 Laboratory Animal Center, China Medical University, Shenyang, Liaoning 110001, P.R. China; 4 Department of Pathology and Pathophysiology Research, China Medical University, Shenyang, Liaoning 110001, P.R. China

**Keywords:** acute myeloid leukemia, P-gp, As_2_O_3_, MDR1

## Abstract

To study the expression of P-glycoprotein (P-gp) and the reversal function of As_2_O_3_, the active ingredient of arsenic, on drug resistance in acute myeloid leukemia (AML) patients, P-gp and cluster of differentiation 34 (CD34) were examined in primary mononuclear and resistant cells, with or without As_2_O_3_. In addition, multidrug resistance gene 1 (*MDR1*) mRNA expression was investigated in K562/D cells and AML patients. In total, 28.6% of newly-treated (NT) patients and 59.1% of relapsed/refractory (RR) patients were P-gp_function_^+^, and 31.7% of NT patients and 59.1% of RR patients were CD34^+^. The positivity rate of P-gp_function_ and CD34^+^ expression in the RR group were significantly higher compared with that in the NT group (P<0.05). In addition, higher CD34^+^, P-gp_expression_^+^ and P-gp_function_^+^ values were observed in older patients compared with younger patients. *MDR1* expression was downregulated in certain patients following treatment with AS_2_O_3_. In the present study, the overexpression of P-gp was the primary cause of drug resistance in the AML patients, and *MDR1* expression was downregulated by As_2_O_3_ in primary leukemia and drug-resistant cells.

## Introduction

In general, the treatment of leukemia consists of chemotherapy and bone marrow transplantation. Clinically, 60–80% of acute myeloid leukemia (AML) patients experience complete remission following routine chemotherapy treatment (1), however, numerous patients relapse. The proportion of relapsed/refractory (RR) patients increases with age (2). Gao *et al* observed that the expression of P-glycoprotein (P-gp) in RR patients was higher than that in treatment-sensitive patients (3). Therefore, the overexpression of P-gp is considered to be the primary cause of multidrug resistance (MDR) in patients with AML. P-gp functions as an ATP-dependent membrane protein, and is principally expressed in excretory tissues located in the placenta, kidneys, liver, intestines, blood brain barrier and blood testes barrier. P-gp is involved in the absorption, distribution and excretion of drugs, xenobiotics and endogenous compounds (4). At present, research into the reversal of MDR in leukemia is primarily aimed at identifying reversal agents that target ATP-binding membrane proteins. Verapamil (VRP) and cyclosporine A (CsA) have been extensively studied in the clinic and the laboratory as potential reversal agents. The therapeutic mechanism of VRP for MDR is the competition with anticarcinogens for the associated binding sites of P-gp, which increases the retention of anticarcinogens in cells (5). CsA, and its derivative, PSC-833, can bind to P-gp in a competitive manner to prevent MDR (6). However, severe side-effects are a common feature of these reversal agents, therefore, their clinical application is restricted. Emerging biotechnologies such as small interfering RNA and P-gp antibodies, which target the function and expression of P-gp, have been successful, but are currently in the early stages of research (7,8). During the differentiation of leukemia cells, CD34 is expressed in AML malignant cells, while the expression P-gp is decreased (9,10). Whether CD34 levels correlate with the expression or function of P-gp remains unclear (11). In recent years, the majority of studies have focused on the treatment of tumors. Arsenic, also known as arsenic trioxide in China, is a traditional Chinese medicine that has been used for >2,000 years. The active ingredient of arsenic, As_2_O_3_, was used for the treatment of psoriasis, rheumatism, leukemia, syphilis and hemorrhoids several centuries ago. The first report of As_2_O_3_ therapy for acute promyelocytic leukemia (APL), a subtype of AML, was in the early 1970s. A research group from the Harbin Medical University of China (Harbin, Heilongjiang, China) identified that intravenous infusions of Ailing-1, a crude solution of As_2_O_3_ and trace amounts of mercury, could be used as a treatment for APL (12–14). Subsequently, researchers at the Shanghai Institute of Hematology (Shanghai, China) demonstrated that As_2_O_3_ could induce APL cell apoptosis and differentiation (15–17). Currently, As_2_O_3_ is used extensively as a treatment for APL and solid tumors in the clinic. A Chinese study that investigated the mechanism of As_2_O_3_ for the treatment of APL was published in 2010 (18). The results revealed that As_2_O_3_ upregulates the degradation of an oncogenic protein involved in APL cell proliferation. In addition, other previous studies have demonstrated that As_2_O_3_ is effective in the treatment of cancer stem cells, and reverses drug resistance in tumor cells (19,20). However, the mechanism behind this action of As_2_O_3_ remains unclear. At present, AML is only treated with As_2_O_3_ when chemotherapeutics are deemed ineffective. Furthermore, As_2_O_3_ is not used as a resistance reversal agent in clinical treatment. The present study aimed to investigate the expression and functional changes of P-gp in AML cells, in order to reveal the role of As_2_O_3_ in the reversal of drug resistance in leukemia cells.

## Materials and methods

### Cell culture and patient samples

K562/D and K562/S drug-resistant cells were maintained in RPMI 1640 medium (GIBCO, Los Angeles, CA, USA) with 10% fetal bovine serum, 100 U/ml penicillin and 100 μg/ml streptomycin, in a humidified atmosphere at 37°C with 5% CO_2_. The primary mononuclear cells were separated from nine AML patients and maintained in RPMI 1640 medium in the conventional manner (21). The primary mononuclear and K562/D and K562/S cells were divided into two groups; a medication group receiving 1μM As_2_O_3_ and a control group receiving no As_2_O_3_. In total, 85 AML patients (59 males and 26 females) from the First Affiliated Hospital of China Medical University (Shenyang, China), and the Department of Hematology in the 202 Hospital of the People’s Liberation Army of China (Shenyang, China), were included in the present study. The mean age of the patients was 41.8 years (range, 18 to 79 years). The present study was performed in accordance with the ethical standards of the 1975 Declaration of Helsinki, as revised in 2000, and was approved by the Institutional Review Boards of the aforementioned hospitals. Written informed consent was obtained from all patients.

### Reagents

Rhodamine 123 (RH123) and CsA were purchased from Sigma-Aldrich (St. Louis, MO, USA). The mouse anti-human monoclonal antibodies against UIC2 and cluster of differentiation 34 (CD34) were purchased from Immunotech (Marseille, France). TRIzol and Lipofectamine 2000 transfection reagents were purchased from Invitrogen (Carlsbad, CA, USA). The diethylpyrocarbonate and Reverse Transcription System were obtained from Promega (Madison, WI, USA).

### Separation of peripheral blood mononuclear cells

In total, 85 AML patients consisting of 2, 8, 37, 15, 19, 3 and 1 case/s were classified with the M0, M1, M2, M4, M5, M6, and M7 AML subtypes, respectively according to the French-American-British classification system (22). The patients were divided into two groups, consisting of either newly-treated (NT) patients (63 cases) or RR patients (22 cases). Samples of 3–5 ml of blood or marrow (immature leukemia cells >70%) were collected from the patients. The mononuclear cells were prepared using the Ficoll-Hypaque technique. Certain cells were used for detecting the expression of P-gp and CD34 by immunocytochemistry, and others were used to detect the function of P-gp.

### Expression of P-gp and CD34 detected by immunocytochemistry

The K562/S and K562/D cells were fixed by incubation in cold acetone for 10 min, and then incubated with 20 μl of mouse anti-human monoclonal JSB-1 (Maixin Biotechnology, Fuzhou, China) and mouse anti-human monoclonal CD34 antibodies (Immunotech) (5 μg/ml) at 4°C overnight. The following day, the biotin-conjugated rabbit anti-mouse IgG secondary antibody (Maixin Biotechnology) and alkaline phosphatase-conjugated streptavidin complex were added, and the samples were incubated at 37°C for 30 min. The samples were then stained with hematoxylin for 2 min, and the slides were examined using an optical microscope. The cells positive for the expression of P-gp and CD34 exhibited a red color in the cell membrane and cytoplasm, and the cells negative for P-gp and CD34 expression were counterstained blue. Positive P-gp and CD34 expression was confirmed by a positive cell number of >20% (in 500 cells).

### P-gp function detected by flow cytometry (FCM)

The leukemia cells isolated from the AML patients, and the K562/D cells, were divided into two groups, receiving either treatment with 200 ng/ml RHl23 and 5 μg/ml CsA or treatment with 200 ng/ml RHl23 only, as a control. Subsequent to the incubation of all cells with RH123 at 37°C for 60 min, 10,000 cells were analyzed by FCM (FACScan; Becton Dickinson, Franklin Lakes, NJ, USA) at a wavelength of 490 nm. An accumulation of >30% indicated a positive result.

### Expression of P-gp in patients treated with As_2_O_3_

In three cases, the patients were treated with a dose of 10 mg As_2_O_3_ daily. The mononuclear cells were isolated from the peripheral blood or bone marrow of the patients following As_2_O_3_ treatment for five days. The total RNA was isolated by TRIzol reagent according to the manufacturer’s instructions (Invitrogen), and cDNA was reverse transcribed from the isolated mRNA using an AMV RNA PCR kit (Takara Bio, Inc., Shiga, Japan), in line with the standard operating procedure. The *MDR1* mRNA primers and probe were as follows: Forward, 5′-CCCTTCAGTGGCTGGTACAT-3′ and reverse, 5′-ACCGCGATATTGATCTCCAC-3′; and TaqMan probe, 5′-FCCGATCCATGCTCAGACAGGATGTGAP-3′. Each polymerase chain reaction (PCR) (25 μl) contained 5 μl 5× buffer, 0.5 μl MgCl_2_ (250 mmol/l), 0.75 μl dNTPs (10 mmol/l), 1 μl TaqMan primers (10 μmol/l, each), 0.6 μl probe (5 μmol/l), Taq polymerase (1.25 units, ABI; Life Technologies, Paisley, UK), 1 μl cDNA sample and 14.9 μl ddH_2_O. The PCR parameters were as follows: 50°C for 2 min and 94°C for 2 min, followed by 40 cycles at 94°C for 15 sec and 60°C for 40 sec. The standard recombinant plasmid was diluted into five gradients and constructed as the reference standard, and the PCR without a patient sample was used as the negative control. The results were analyzed using the standard software provided with the ABI 7900HT Fast Real-Time PCR System (Life Technologies). All measurements were performed at least three times.

### Statistical analysis

Statistical analysis was performed using SPSS 17.0 (SPSS, Inc., Chicago, IL, USA). Statistical significance was determined by a one-way analysis of variance and Student’s t-test. Correlation between CD34 expression, P-gp expression and patient age was analyzed by Spearman’s rank correlation. P<0.05 was considered to indicate a statistically significant difference.

## Results

### Expression of CD34 and P-gp, and functional analysis of P-gp in AML

**Figure 1:**
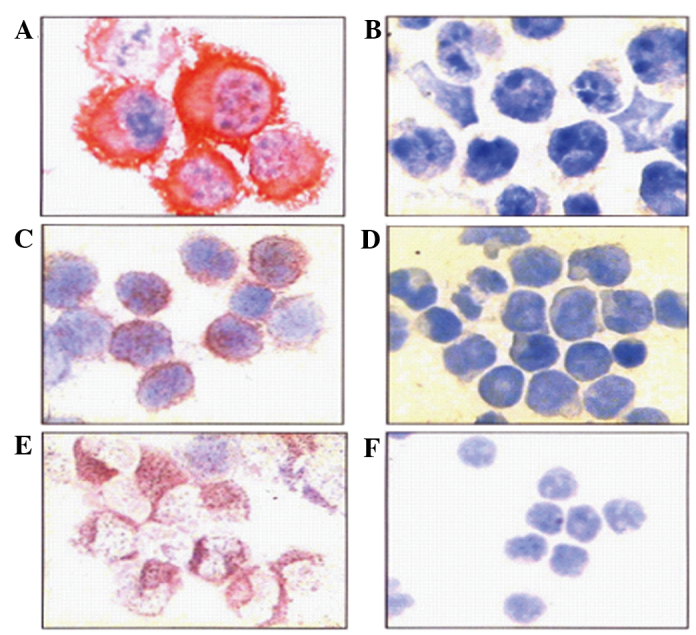
Immunocytochemical fluorescence results showing the expression of cluster of differentiation 34 (CD34) and P-glycoprotein (P-gp) in the (A) K562/D and (B) K562/S cells. Red indicates the expression of P-gp and blue indicates the counterstain of the nucleus. The (C) positive and (D) negative expression of P-gp in the AML cells, and the (E) positive and (F) negative expression of CD34 in the AML cells is also shown. AML, acute myeloid leukemia.

**Figure 2:**
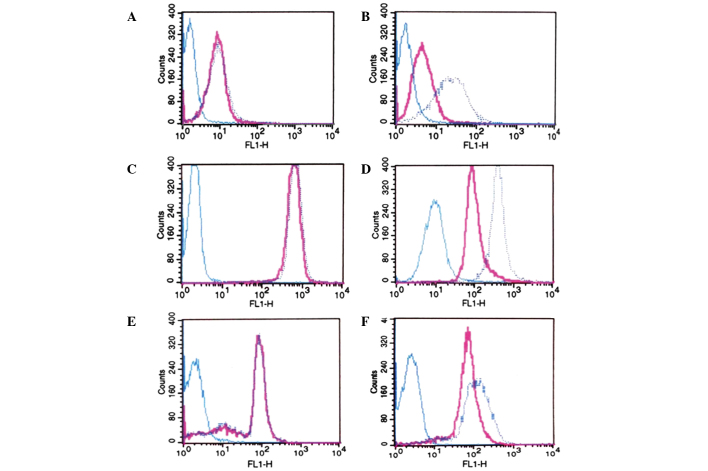
Expression and function of P-gp detected with flow cytometry. The expression of P-gp in the (A) K562/S and (B) K562/D cells, and the expression of P-gp function in the (C) K562/S and (D) K562/D cells. (E) Negative and (F) positive P-gp function observed in the AML cells of the patients. (A–D) In the expression analysis, the blue solid line is the blank control, the pink peak is the secondary antibody only (negative control) and the blue dashed line indicates the experimental group. (E and F) In the functional analysis, the blue solid line is the blank control, the blue dashed line indicates the cells that were treated with RH123 and cyclosporin A, and the pink line indicates the negative control (cells that were only treated with RH123). P-gp, P-glycoprotein; AML, acute myeloid leukemia.

The expression and function of P-gp in the leukemia cells from the AML patients was assessed using immunocytochemistry (Figs. 1 and 2). In total, 23 of 63 (36.5%) NT patients and 11 of 22 (50%) RR patients were P-gp_expression_^+^. However, no statistically significant difference was identified between the two groups. Furthermore, 18 of 63 (28.6%) NT patients and 13 of 22 (59.1%) RR patients were P-gp_function_^+^. The positivity rate of P-gp_function_ was significantly higher in the RR group than in the NT group (P<0.05). In addition, P-gp_expression_ demonstrated a positive correlation with P-gp_function_ (P=0.0001, r=0.579).

In order to analyze the correlation between CD34 and P-gp expression, the expression of CD34 was investigated using immunocytochemistry (Fig. 1E and F). The results demonstrated that 20 of 63 (31.7%) patients in the NT group, and 13 of 22 (59.1%) in the RR group, were CD34^+^. The expression of CD34 in the RR group was significantly higher than that in the NT group (P<0.05). In addition, CD34 expression was positively correlated with P-gp_function_ (P=0.0001, r=0.579) and P-gp_expression_ (P=0.0001, r=0.483).

To analyze the correlation between CD34 expression, P-gp expression and function, and patient age, a total of 85 AML patients were divided into two groups, consisting of patients either <50 years old or ≥50 years old. The results revealed that 8 of 46 (17.4%), 12 of 46 (26.1%) and 9 of 46 (19.6%) NT patients in the <50 years group were CD34^+^, P-gp_expression_^+^ and P-gp_function_^+^, respectively. Furthermore, 11 CD34^+^ cases (64.7%), 11 P-gp_expression_^+^ cases (64.7%) and nine P-gp_function_^+^ cases (52.9%) were found in 17 NT patients from the ≥50 years group. Notably, those patients ≥50 years had a significantly higher CD34^+^, P-gp_expression_^+^ and P-gp_function_^+^ values compared with those <50 years (P<0.05).

### Effect of MDR1 mRNA in K562/D cells and AML patients treated with As_2_O_3_

Out of a total of 85 AML patients, *MDR1* mRNA was detected in three patients positive for P-gp expression following As_2_O_3_ treatment, and in nine cases of primary mononuclear leukemia cells following As_2_O_3_ treatment. The quantitative PCR results demonstrated that the expression of *MDR1* mRNA in the three patients was significantly downregulated following treatment with As_2_O_3_ (P<0.05; Table I). Significant downregulation of the *MDR1* mRNA expression was also observed in eight cases of primary mononuclear leukemia cells following treatment with As_2_O_3_ (P<0.05). Furthermore, the *MDR1* mRNA expression level decreased 2.3-fold in the K562/D cells following treatment with As_2_O_3._

### Effect of P-gp expression and function in K562/D cells following treatment with As_2_O_3_

Using FCM, it was observed that the expression of P-gp decreased following four days of treatment with 1μM As_2_O_3_. The fluorescence intensity of P-gp decreased by 30.5%, whereas the intensity of RH123 increased by 38.1% (P<0.05). Therefore, it was concluded that As_2_O_3_ could prolong drug release in the K562/D cells by inhibiting the expression of P-gp and attenuating drug elimination, which is attributable to the function of P-gp.

## Discussion

The present study identified that P-gp, involved in drug resistance, was expressed in AML cells, and that As_2_O_3_ could reverse the drug resistance in P-gp-expressing leukemia cells. A previous study demonstrated that the expression of P-gp in RR AML patients was higher than that in NT patients (23). However, using FCM, the present study identified extremely few cases of positive P-gp expression (data not shown). P-gp_function_, however, was found to be higher in RR patients than in the NT patients. The expression of P-gp, detected by immunocytochemistry, demonstrated no significant difference between the two patient groups, however, P-gp_expression_ was revealed to be positively correlated with P-gp_function_. Therefore, it was hypothesized that immunocytochemistry and FCM may not have been sensitive enough to detect the expression of P-gp in the present study.

Previous studies identified that leukemia cells exist as differentially-phased subgroups of cells (24–26). Certain leukemia cells can reproduce and have long-term proliferation abilities. Others possess a finite capacity to replicate and eventually differentiate into immature leukemia cells, which account for the majority of malignant cells; these express the CD34 in the AML subgroup cells (1). The positivity rate of P-gp expression, and the function of P-gp, demonstrated a downward trend during the CD34^+^/CD33^−^, CD34^+^/CD33^+^ and CD34^−^/CD33^+^ cell maturation processes. The present study identified that the expression of CD34 was positively correlated with P-gp_function_ and P-gp_expression_. Furthermore, CD34 had a higher P-gp positivity rate in the RR patient group. Therefore, it was concluded that the reason for the increase in the P-gp positivity rate observed in the RR patient group was due not to induction by chemotherapeutics, but to the elimination of leukemia cells that were sensitive to treatment, which allowed the CD34^+^/P-gp^+^ subgroup of cells to preferentially proliferate. Therefore, it is important to determine whether it is feasible to use an inhibitor of P-gp during the initial treatment with chemotherapy, so as to eliminate the malignant clone of P-gp^+^ cells and avoid MDR caused by long-term chemotherapy. A previous study (27) found that the positivity rates of P-gp expression and function increased with increasing patient age. In the present study, it was revealed that the expression of CD34 and P-gp, and the function of P-gp, were significantly different between the <50-year-old and ≥50-year-old patient groups. In addition, older patients demonstrated an increased P-gp positivity rate. Older patients are often treated with lower doses of drugs in order to reduce side-effects and complications in clinical therapy. Unfortunately, these lower doses can affect treatment outcomes and prognosis. Therefore, it will be critical to detect the function of P-gp as early as possible in order to provide a rational and efficacious treatment.

Previous studies have demonstrated that As_2_O_3_ could reverse anticancer drug resistance by inhibiting the expression of P-gp (28,29). The present study investigated the expression of *MDR1* using quantitative PCR prior to and subsequent to treatment with As_2_O_3_ in three RR AML patients. The expression of *MDR1* was inhibited by As_2_O_3_ *in vivo*. Due to the small number of As_2_O_3_ treatment cases in clinical therapy, primary mononuclear leukemia cells positive for P-gp expression were cultured from nine AML patients and analyzed for changes in *MDR1* expression following treatment with As_2_O_3_. According to quantitative PCR, the expression of *MDR1* was significantly downregulated following treatment with As_2_O_3_.

The drug-resistant cell line used in the present study, K562/D, was induced by Adriamycin (ADM). This cell line has typical MDR characteristics and is resistant to a number of chemotherapeutics, such as ADM, daunorubicin and vincristine (VCR). Compared with K562 wild-type cells, K562/D cells possess ~160 and 500 times greater drug resistance to ADM and VCR, respectively (30). Therefore, the K562/D cell line is an appropriate model to study the role of P-gp expression in AML drug resistance. In the present study, the expression and function of P-gp were significantly downregulated in the K562/D cells following As_2_O_3_ treatment, as detected by FCM. The K562/D cell line has commonly been used to examine the function of the P-gp ion pump (31). The overexpression of P-gp can result in a lower drug concentration in the cells, and as observed in the present study, the RH123 fluorochrome is pumped out the cells as the substrate. The experimental results demonstrated that As_2_O_3_ could inhibit the expression of P-gp in primary mononuclear and drug-resistant cells. The results of the present study demonstrate that As_2_O_3_ has the potential to reverse drug resistance, and therefore should not only be used when chemotherapeutics prove ineffective, but also as a resistance reversal agent used in coordination with other chemotherapeutics. These results provide support for the therapeutic development and application of As_2_O_3_. The mechanism of As_2_O_3_-mediated P-gp inhibition warrants further investigation.

## Figures and Tables

**Table I:** Expression of *MDR1* mRNA before and after As_2_O_3_ treatment.

	*MDR1* mRNA expression	
	
Samples	Before	After
1	5.7×10^−3^	3.8×10^−3^
2	2.5×10^−4^	2.4×10^−4^
3	8.0×10^−2^	1.4×10^−2^
4	9.0×10^−4^	3.7×10^−4^
5	1.3×10^−3^	1.2×10^−3^
6	1.7×10^−3^	7.8×10^−3^
7	2.2×10^−3^	3.7×10^−3^

MDR1, multidrug resistance gene 1.
